# Temporally Local Tactile Codes Can Be Stored in Working Memory

**DOI:** 10.3389/fnhum.2022.840108

**Published:** 2022-05-27

**Authors:** Arindam Bhattacharjee, Cornelius Schwarz

**Affiliations:** ^1^Werner Reichardt Center for Integrative Neuroscience, Systems Neuroscience, Eberhard Karls University Tübingen, Tübingen, Germany; ^2^Hertie Institute for Clinical Brain Research, Eberhard Karls University Tübingen, Tübingen, Germany

**Keywords:** tactile code, working memory, psychophysics, fingertip, human

## Abstract

Tactile exploration often involves sequential touches interspersed with stimulus-free durations (e.g., the time during which the hand moves from one textured surface to the other). Whereas it is obvious that texture-related perceptual variables, irrespective of the encoding strategy, must be stored in memory for comparison, it is rather unclear which of those variables are held in memory. There are two established variables—“intensity” and “frequency”, which are “temporally global” variables because of the long stimulus integration interval required to average the signal or derive spectral components, respectively; on the other hand, a recently established third contender is the “temporally local” variable that codes for kinematic profiles of very short, suprathreshold events in the vibrotactile signal. Here, we present the first psychophysical evidence that temporally local variables can be stored in memory. To that end, we asked participants to detect changes in pulsatile indentation stimuli at their fingertips with and without a gap of 1 s between stimulus presentations. The stimuli either contained global variables alone (change of pulse rate), or a mix of local and global variables (change of pulse shape). We found, first, that humans are much better at detecting a change in stimuli when local variables are available rather than global ones alone—as evident by the fact that 21 compared to only 6 participants out of 25 yielded a valid psychophysical curve, respectively. Second, this observation persists even when there is a gap between the stimuli, implying local variables must be stored in memory. Third, an extensive array of relevant intensity definitions failed to explain participants’ performance in any consistent manner, which implies that perceptual decisions were less likely to be driven by intensity coding. Taken together, our results suggest that humans perform pulsatile change detection utilizing local pulse shape, and to a lesser degree global pulse rate, and that both parameters can be stored in memory.

## Introduction

The movement of our fingertips on textured surfaces generates skin vibrations (i.e., vibrotactile stimuli), which are captured by the tactile receptors and then processed by the brain. To accomplish decisions that play out on a large time scale, vibrotactile information frequently must be held in memory. This is also true in a simple case when two textures are touched in a sequence—for example, to compare one against the other on some qualitative aspect. The vibrotactile information of the first texture must be held in memory at least until the next texture is touched. There have been many studies that investigated, in detail, various aspects of vibrotactile perception and working memory in rats (Fassihi et al., 2014), humans (see for review Preuschhof et al., 2006; Pleger and Villringer, 2013), and non-human primates (see for review Romo and Salinas, 2003). However, since different vibrotactile coding principles capture different features of a vibrotactile stimulus, it is not clear which of those features are stored in the memory. The investigation of this question is the objective of the current study.

Two principal candidate tactile coding schemes are discussed here. The classical one is thought to average the signal over a *long* stimulus period (hundreds of milliseconds) either by summing the signal itself (“intensity”) or by summing spectral components (“frequency”). We call this strategy “temporally global”. In contrast, the second is a novel coding scheme that analyses the kinematics of the signal (its “shape”) to detect events quasi instantaneously—within a few milliseconds. We call this strategy “temporally local”. The idea that local variables may play a decisive role in tactile perception comes from studies of naturalistic vibrotactile stimuli evoked by touching textured surfaces. Vibrotactile signals in this situation are characterized by frictional processes that contain short stick-slip events, which carry texture information coded in their kinematic shape. This insight has been strongly supported by studies in the rodent whisker system, where the biomechanics of whiskers (i.e., the sensor) has been studied in detail using high-resolution videotaping (Ritt et al., 2008; Wolfe et al., 2008; Oladazimi et al., 2018, 2021). In this model system it has been established that stick-slip events (or short events in stimulated trajectory waveforms) specifically trigger neural activity in the ascending tactile pathways (Jadhav et al., 2009; Chagas et al., 2013; Waiblinger et al., 2015a, b; Laturnus et al., 2021). Similarly, in the human glabrous skin, there is evidence that frictional events are generated when the fingertip moves on textured surfaces, and that such frictional events evoke activity in the ascending tactile pathways (Westling and Johansson, 1987; Prevost et al., 2009; Delhaye et al., 2014, 2021; Schwarz, 2016).

While tactile interaction of textured surfaces is informative to learn about possible coding schemes, theories of human tactile perception have been most efficiently tested by studying the detectability/discriminability of indentation stimuli, which enable precise quantification of the features contained in the vibrotactile signal. Experiments using vibrotactile indentation stimuli led to estimates of the length of the integration window needed to extract the global coding variables like “intensity”, and “frequency” (Luna et al., 2005). The insight that short frictional events in the vibrotactile signal could capture a large fraction of available texture information gave rise to the alternative notion of temporally local coding, i.e., extraction of kinematic shape. A defining property of this read-out is its ultra-short integration time window down to a few milliseconds (Schwarz, 2016) compared to the long integration time windows required to determine the global signal variables. Recently, Bhattacharjee et al. (2021), using pulsatile indentations at the fingertip, provided evidence that the human tactile system is highly sensitive to maximal kinematic parameters of indentation pulses, and that it utilizes these local coding variables to perform perceptual decisions. This finding is similar in many ways to that reported in the rat whisker-related tactile system (Waiblinger et al., 2015a, b), pointing to frictional bases of tactile perception, although the biomechanical details of the human fingertip are certainly different from that of the whisker system.

Regardless of the encoding strategy used by the tactile system, it is obligatory that the sensory information is stored in memory. Therefore, it is an important question to clarify which coding variable can be stored and whether performance deteriorates if the perceptual process model contains elements of working memory. Previous studies demonstrated that tactile stimulus information can be kept in memory for many seconds in rodents and humans (Gerdjikov et al., 2010; Fassihi et al., 2014). However, without contemplating the possibility that local codes may be used, these studies suggested that mean speed (one of several formulations of intensity, see Bhattacharjee et al., 2021) matches the behavioral performance the best, and consequently is the feature that is stored in memory. The discovery of local variables as a candidate feature for encoding vibrotactile stimuli (Waiblinger et al., 2015a, b; Bhattacharjee et al., 2021), warrants a re-examination of the question of which variable is stored in memory, and opens the possibility for a coding and storing strategies specific to the tactile system—namely, that critical texture information is compressed by storing local features contained in rare short frictional events. In the current study, we investigated this possibility by introducing a stimulus-free gap of 1 s duration between a pair of presented stimuli to tap into the well-characterized time span of human working memory (500 ms to several seconds; Burton and Sinclair, 2000). We implemented a detection of change task using pulsatile vibrotactile skin indentations. The pulsatile stimuli can either change in pulse “rate” or “shape”, a characteristic that had been used before to delineate local and global coding schemes (Bhattacharjee et al., 2021).

## Methods

### Participants

Twenty-five neurologically healthy (self-reported) participants (age: 20–50 years, median age 25 years; 12 female), all right-handed (determined based on questions modified from the Edinburgh Handedness Inventory; Oldfield, 1971), participated in four experimental conditions. Counterbalancing these conditions yielded 24 (factorial of 4) unique testing sequences, which we assigned to participants sequentially as they joined the participants’ pool; two participants were tested on one such sequence by error, we decided to include both participants’ data in the study. The participant recruitment process discouraged individuals from signing-up if they have been diagnosed with any disorders that may affect their tactile acuity—for example, dyslexia (Grant et al., 1999; Laasonen et al., 2001), diabetes (causes peripheral neuropathy and action potential conduction delays; Hyllienmark et al., 1995), learning disabilities, or even calluses to the left index fingertip (the tested finger). Our institutional research ethics board approved the study; participants signed the informed consent and received payment for their participation.

### Vibrotactile Stimulation

The equipment used to deliver the stimuli was identical to that reported in our recent study (Bhattacharjee et al., 2021). A plastic circular disc of 2.9 mm diameter, attached to a galvo-motor (model 6220H, Cambridge Technology, USA), was used to apply the vibrations to the distal pad of the left index fingertip. An in-house designed amplifier drove the galvo-motor that generated high-precision displacements; the galvo-motor was calibrated using a highly sensitive (micron resolution) laser distance estimator. A personal computer equipped with an I/O board (PCI-MIO-16E-1 I/O, National Instruments, Austin, USA) running Matlab (Natick, USA) was used to conduct the experiment—i.e., generating the stimulus waveforms, controlling the galvo-motor movements, assessment of participant responses, and implementation of the experimental schedule. Participants’ perceptual decisions (Yes/No) were delivered by pressing one of two buttons on a wireless presenter’s clicker (R400 Logitech Presentation Remote, Lausanne, Switzerland) with their right hand. Voltage waveforms controlling the movements of the galvo-motor were digitized at a resolution depth of 12 bits and a rate of 40,000 samples per second.

The participant’s arm rested on a height-adjustable platform, and their left index finger was clamped in a finger housing using two fixtures, a ridge that secured the front rim of the nail, and a double-sided tape that affixed the back of the fingernail to the ceiling of the housing. After ensuring that the testing finger was securely positioned and immobilized, an adjustable tri-axis micromanipulator was used to attach the circular disc of the galvo-motor to the distal pad of the testing finger. The disc first coarsely approached the skin until the participant reported touch-down. It was then carefully moved further to reach the starting point of pulsatile stimuli of 1 mm. Pulsatile stimuli engaged the skin from that base toward further indenting positions. During the experiment, the testing region of the fingertip was in contact with the circular disc, and no other part of the galvo-motor touched any part of the participants’ finger. To avoid the possibility of their fingernail touching any part of the stimulator, the participants were requested to trim their nails. The arm platform as well as the galvo-motor platform sat on two anti-slip anti-vibration mats that were not touching each other.

All stimuli delivered in this study were pulsatile, i.e., series of pulsatile indentations separated by rest, at a base indentation of 1 mm, identical to the ones used before (Bhattacharjee et al., 2021). One trial included two such pulse trains—the reference, and the comparison. The reference stimulus, in all the experimental sessions, was a 500 ms pulse train presented at a pulse rate of 90 Hz. The individual pulses were composed of single-period sinusoids smoothly setting off from the base indentation (i.e., to generate the pulse waveform, a sinusoid starting from one minimum and ending at the next was used). The single pulses were extracted from sinusoids at a frequency of 170 Hz and an amplitude of 40 microns. The comparison stimulus (also a 500 ms pulse train), either changed in terms of pulse rate (90–135 Hz in steps of 5 Hz, pulse shapes were the same as the reference pulses) or in terms of pulse shape [the sinusoid used to construct the pulses changed from 170 to 240 Hz in steps of 5 Hz, i.e., only the width of the pulses changed from 5.882 ms (1 s/170 Hz) to 4.167 ms (1 s/240 Hz)]. These stimulus ranges are identical to the ones, for which psychometric curves already had been reported (Bhattacharjee et al., 2021). As before, the aim was to engage the Pacinian primary afferent system—to that end, the pulse width was chosen to relate to those relevant frequencies that would maximally activate the Pacinians (Freeman and Johnson, 1982, 5.882 ms translate to 170 Hz, and 4.167–240 Hz waveform frequency of the sinusoidal pulse). The choice of the upper extreme (240 Hz) of the waveform frequency was additionally constrainted by the mechanical limits of the galvo-motor actuator (i.e., absence of resonant oscillations).

In all sessions, each stimulus level was presented 30 times, resulting in a total of 840 trials spread over three testing blocks (i.e., 280 trials per block) in shape-change sessions, and 540 trials spread over three testing blocks (180 trials per block) in rate-change sessions. Note that shape-changes induce changes in both local and global coding variables, while rate-changes induce exclusively global changes (see “Results” Section).

In addition to “frequency cues”, the other key temporally global tactile variable is “intensity”. Intensity was calculated across the entire 500-ms stimulus duration and was formulated as “mean absolute velocity” (mean speed), “mean squared velocity”, “mean absolute velocity taken to the power of three”, “mean absolute acceleration”, “mean squared acceleration”, and finally “mean absolute velocity taken to the power of three”. The reason for these choices is described in detail elsewhere (Bhattacharjee et al., 2021). In a nutshell, human participants show relatively poor change detection only in a limited portion of the stimulus range spanned by changes in pulse widths and amplitudes (namely in a range where these two parameters change in opposing directions). The mentioned six intensity formulations show iso-feature lines (i.e., lines through stimulus range, on which the corresponding intensity does not change at all) that evenly cover the range of poor performance. The reasoning is that the absence (or near absence) of a stimulus cue in a range of stimuli characterized by poor responses may well point to a perceptual role of these cues (while stimulus ranges giving rise to superior psychophysical performance have much lower potential to disentangle the perceptual role of the mapped stimulus cues).

### Perceptual Tasks and Psychophysics Procedure

Each participant was tested on each change condition (shape and rate) presenting the reference and comparator stimuli either seamlessly (no gap), or separated by 1 s (gap), resulting in four different tests (four sessions). Always, 50% of the total trials used identical reference and comparator stimuli (“no change”), while the other 50% present the different change-shapes (“change”). The instruction was “Did you perceive a change in the stimulus?”. The participants’ responses (Yes/No) were recorded after each trial. “Yes” responses after “change” trials and “No” responses after “no-change” trials were considered as correct responses. In this study, we aimed for a measurement of perceptual precision. Therefore, to avoid a possible speed-accuracy trade-off, we explicitly instructed the participants to respond as accurately as possible, rather than as fast as possible.

Prior to starting each session, the experimenter read out loud the instructions for the respective session and the participant was encouraged to ask questions about the task, should they have any; next, to ensure that the participant understood the task, we asked the participant to repeat the task instructions back to us, and the session began only after we were satisfied that the participant had fully understood the task. At the beginning of the first block in each testing session, participants received 20 practice trials (randomly presented, 10 of each type—change and no change) to get acquainted with the experimental setup and the procedure. After each response, in the practice as well as in the testing phase, participants heard a feedback tone delivered through headphones informing them about the trial’s result (correct/incorrect). Aside from the auditory feedback, at the end of each block, participants also saw their performance score as the total percent correct for that block. In all testing sessions, an intertrial interval of 5 s duration started after the participant responded. A 2-min break was imposed after each block during which the participants were encouraged to stand up and walk around, the break lasted longer if the participant so wished. Throughout the session, a white noise was played out loud next to the tactile stimulator to mask any movement-related noise of the actuator.

### Statistical Analysis

To investigate whether participants can retain local feature information during the gap duration (i.e., in their working memory) we required the participants to perform well in the task such that we could generate their psychometric function for each task (see subsection “Perceptual Task”). However, the responses of four participants in the shape-change detection task (that did not require the involvement of the working memory) did not yield a psychometric function; intriguingly, these participants were also unable to perform well enough in the corresponding gap-condition resulting in their failure to generate a psychometric function. Data from these four participants were not considered for the data analysis.

To quantify each participant’s performance (proportion reported “change”) in each session, we used a dedicated analysis software—*psignifit* (Wichmann and Hill, 2001; Ver. 3; available at http://psignifit.sourceforge.net/). The program fits a mixture model, a cumulative normal psychometric function, of the following form:


P(x;m,w,γ,δ)=γ+(1−δ−γ)S(x;m,w)

where *P* is the psychometric function, *S* a sigmoidal function (here a cumulative Gaussian), *x* the stimulus level (i.e., pulse width, or pulse rate), *m* the value at which *S*(*x*) = 0.5 (threshold), *w* the width of the sigmoid (the interval [*x*1, *x*2] where *S*(*x*1) = 0.05 and *S*(*x*2) = 0.95), γ the false alarm rate (lower asymptote), and δ the lapse rate (upper asymptote). The psignifit algorithm computes the maximum likelihood of *P* generated from combinations of [*m*, *w*, *γ*, *δ*]. Only psychometric functions *P* that pass *P*(*x*) = 0.5, (i.e., yielded a threshold) were considered as valid performance, all others were classified as invalid and not further analyzed. Further, to compensate for the false alarms, we performed false alarm correction (Klein, 2001) and then considered the x-axis value at *P* = 0.5 as the threshold for all statistical analyses.

## Results

**Figure 1:**
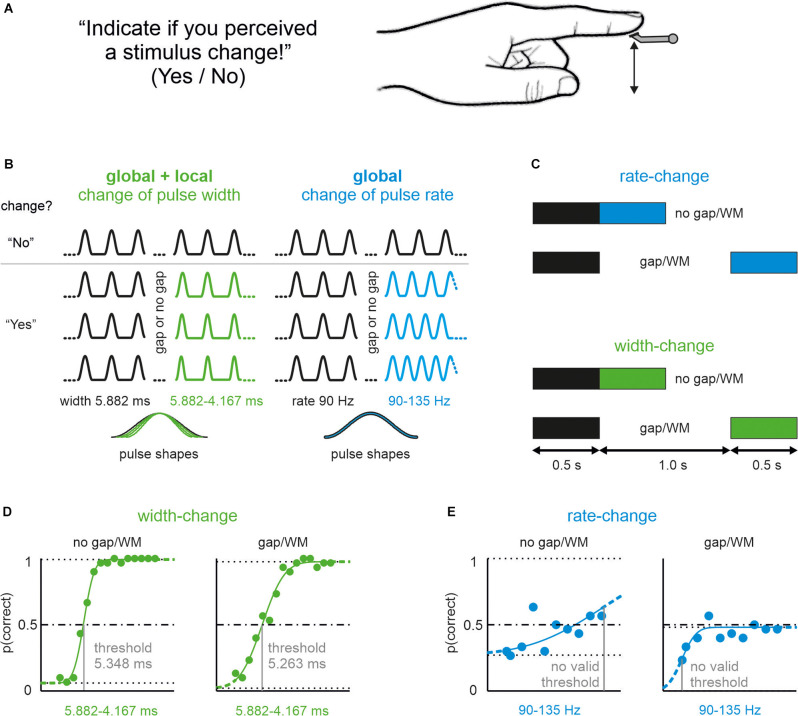
Pictorialdescription of the task, and stimulus manipulations used in thisstudy. **(A)** Participants’ perceptual task and instruction.All participants received vibrotactile stimuli on their left indexfingertip. **(B)** Experimental realization of shape-changes(pulse width) and rate-changes (inter-pulse-duration). In eachtrial, participants received pulsatile stimuli starting with areference stimulus (black) and a comparison stimulus (color; in 50%of the stimuli there was no change, i.e., reference followed byreference again). Example stimuli of trials from two conditionsrepresenting shape-change (green) and rate-change (blue).Above the gray line are stimuli without change (correct answer:“No”), below some example stimuli with a change (correct answer:“Yes”). Bottom: magnified single pulses from each of the fourexample stimuli illustrating pulses without (blue) and with (green)local shape changes. **(C)** Pictorial representation of the Gapvs. no-Gap conditions. In all the experimental conditions, thereference stimulus and the comparison stimuli were 500 ms long. Inthe no-gap conditions, the comparison stimuli seamlessly followed the reference; however, in the gap conditions, the reference and comparison stimuli were separated by an interval of 1,000 ms (in which the stimulator did not move). **(D)** Representative example of the psychophysical performance of one participant on the stimulus set presenting width changes. The no gap (left) and gap experiment (right) is shown. The upper and lower limits, as well as the line indicating p (correct) = 0.5 are shown (dashed horizontal lines). The estimated threshold is indicated by the gray vertical line. **(E)** The same participant’s performance on the rate-change stimulus set. No valid threshold could be assessed (exclusion criteria: 1. curve did not reach *p* = 0.5; 2. threshold was located on the limit of the stimulus range, see “Methods” Section). Note that within the total sample of 25, a minority of participants (only 6 out 25) performed successfully on this stimulus sets as well. Line conventions as in **(D)**.

We tested 25 participants in a vibrotactile change detection task. Pulsatile skin-indenting stimuli were presented at the glabrous skin of the proximal phalanx of the left index finger (Figure 1A). In each trial a reference stimulus of 500 ms duration was given first, followed by either in seamless ways or after a gap of 1 s, a comparison stimulus of the same duration. In half of the presented trials, the comparison stimulus differed from the reference. After each trial participants reported their decision, whether or not they perceived a change by pressing the “Yes” or “No” response buttons. The difference between reference and comparison stimuli came in two variants. Either the shape (width) of pulses or the pulse-rate (inter-pulse interval) was altered (Figure 1B). All participants were tested in four sessions on different days, which presented the four combinations of shape/rate changes and gap/no-gap conditions (Figure 1C). The order of these sessions was different for each participant and was determined by a pseudorandom sequence.

**Figure 2:**
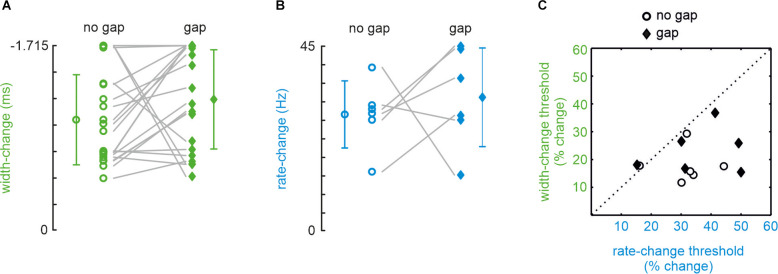
Detectionof change thresholds in shape- (green) and rate- (blue) change experiments. Data points with error bars (SD) indicate mean thresholds in each experimental condition. The ordinates (note the reverse direction) scale the difference of width/rate contained in comparison and reference stimuli. **(A)** Thresholds yielded by 21 (out of 25) participants in the no-gap (circles) and gap (diamonds) conditions of the pulse-shape-change experiment. **(B)** Same for the rate-change experiments. In rate-change experiments, only 6 out of 25 participants generated a psychometric function that crossed the 50% threshold line. **(C)** Thresholds expressed as the percentage change (compared to width and rate of the reference stimulus). The six participants who yielded valid performance in all four combinations of width/rate/no gap/gap conditions are shown (same participants as shown in panel **B**).

The no-gap stimuli were identical to a subset of stimuli presented in a previous publication (Bhattacharjee et al., 2021; in the present study we only used shape-change stimuli on iso-feature-line a, as in their Figure 2; rate-change stimuli, used here and previously, were identical). The local shape was, therefore, varied exclusively by reducing pulse width (and not amplitude) in the present experiments (Figure 1B). A typical experimental result obtained from one participant of our sample of 25 is shown in Figures 1D,E. A majority of 21 out of 25 participants readily detected a change in pulse width (i.e., yielding a psychometric curve crossing a detection probability of 0.5; see “Methods” Section) confirming previous results (Bhattacharjee et al., 2021). Furthermore, introduction of a gap did not significantly impair performance (Figure 2A; paired sample *t*-test; *t*_20_ = 1.492, *p* = 0.151; AUC = 0.11). Rate-stimuli were much harder to discriminate than width-changes: within our population of 25, only six participants yielded valid psychometric curves. The performance of the six successful participants in the gap and no-gap sessions was similar, suggesting that they were able to store the global coding variable used for the performance (Figure 2B; paired sample *t*-test: *t*_5_ = 0.612, *p* = 0.567, AUC = 0.54).The thresholds (now expressed in percentage changed) of the same six participants and for both rate vs. shape manipulations are shown in Figure 2C. While gap and no gap thresholds intermingle, it is evident that the percentage of stimulus change required to reach the threshold is higher in rate- as compared to shape-change detection. These data, obtained from the most successful participants, help to explain why so many participants failed the rate-change experiment as compared to the shape-change experiment. It is important to note that we could not find evidence that certain participants performed generally better at the tasks. Correlations of performance of the six participants (shown in Figures 2B,C) on the four experimental conditions (shape/no gap; shape/gap; rate/no gap; rate/gap) were low and partly negatively correlated (no significance at *p* < 0.05). Similarly, within the 21 participants that performed well in shape-change experiments (shown in Figure 2A), the correlation of performance in no gap vs. gap experiments was low at *r* = 0.13 (*p* = 0.58).

In summary, the superior performance in shape-change vs. the rather poor performance in rate-change sessions pointed to the possibility that participants were using local coding variables. On the other hand, the similarity of the performances in gap vs. no-gap sessions for both types of change, suggested that, whatever they used to encode the stimuli, could also be stored in working memory.

**Figure 3:**
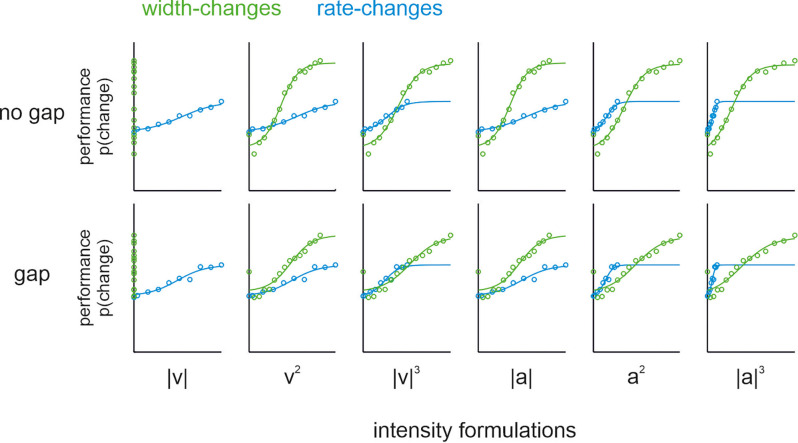
Psychophysical population data and psychometric curves (logistic fits). Comparison of psychometric functions from pulse shape- (green) and rate- (blue) change experiments in no-gap (upper row) and gap (lower row) conditions. Within each gap as well as no-gap conditions, the six graphs replot the same data rescaled to each of the six intensity formulations (columns). The abscissae in each panel refers to the following scaled intensities (summed over the entire comparison stimulus duration): |V| or mean speed, |V|^2^ or squared velocity, |V|^3^ or cubic absolute velocity, |a| or mean acceleration, |a|^2^ or squared acceleration, and |a|^3^ or cubic absolute acceleration. Note, in order to demonstrate the curves along the entire measured range, the abscissa was scaled to the extreme intensities found in the union set of rate- and shape change stimuli. As our stimulus design was guided by shape and rate (cf. Figure 1), the intensities of stimuli contained did not exactly match, and cover slightly different ranges (the difference in range becomes higher with higher powers used to calculate the intensity). For the ease of comparison, the fitted sigmoidal functions are shown across the whole intensity range scaled on the abscissa. Further, note that the performance for shape-change experiments (green) plotted on mean speed (leftmost panels) did not yield a S-shaped psychometric curve. This is trivially so, as stimuli were picked from a set of pulse trains designed to keep this variable constant (i.e., there was no change of mean speed between reference and comparison stimulus).

It is noteworthy that width-changes go along with changes in intensity values (i.e., the stimulus mean is going to be different with different pulse widths). A problem with intensity coding is that the exact formulation of intensity is far from clear. Pulsatile stimuli of sinusoidal shape that change in pulse width exclusively, as used here, are known to keep mean speed (one of the six intensity formulations) constant (Bhattacharjee et al., 2021). To test whether the other five intensity formulations (i.e., mean squared velocity, cubic absolute velocity, mean acceleration, squared acceleration, and cubic absolute acceleration) may play a prominent role, we rescaled the stimuli according to each corresponding intensity formulation. The rationale for rescaling was that if the performance in the shape-change condition is actually due to a change in intensity (defined by one of these formulations), then the psychometric functions of the rate-change condition (which only contains global variables) should match those of the shape-change condition. Performance on the no-gap-sessions confirmed the previous study, as none of the intensity formulations led to an alignment of psychophysical performance, except perhaps the intensity defined as cubic absolute velocity (Figure 3; upper row). However, this particular intensity formulation can be safely excluded to offer an explanation for the performance, as Bhattacharjee et al. (2021) showed the general non-correspondence of psychophysical curves when applying a more varied set of shape-changes than used here. These confirmative results suggest that none of the six relevant intensity formulations alone can explain the performance of our participants. For our present question about the variables stored in memory, the most important fact was that we did not observe any systematic deviation of the no-gap performance from the gap performance (Figure 3; upper vs. lower row).

## Discussion

Here we studied which encoded variables of pulsatile vibrotactile stimuli are stored in memory. To this end, we tested human performance on vibrotactile change detection tasks with stimuli where the change occurred either within a continuous presentation of a train of pulsatile deflections (no-gap-condition), or where a 1-s stimulus-free interval (gap-condition) interrupted the reference and the comparison. To delineate encoded variables, we implemented different stimulus layouts: rate-changes contain exclusively global variables, while shape-changes contain local and global variables. Despite the fact that a far higher proportion of participants were able to detect shape-changes than rate-changes, we uniquely found that introducing the gap did not significantly alter the performance of those that performed well on both tasks. Rescaling the stimuli to reasonable intensity formulations suggested that intensity coding does not play a consistent and dominant role—at least for stimuli that we presented here. Together these results confirm earlier findings that temporally-local shape information as well as global rate information underly perception. Second, they offer a novel insight by showing that stimuli encoded in local as well as global fashion can be stored in memory.

We observed that only 6 out of 25 participants performed on rate-changes well enough to fit a psychometric function. Importantly, all those participants who failed on rate-changes did perform well on the shape-change experiments (in fact overall a majority of 21 out of 25 did), excluding the possibility of a general inability of some individuals to perform on this type of psychophysical test. This finding firstly argues in favor of the notion that shape and rate cues are both encoded. Secondly, it points to a dominance of local shape coding over global rate coding. Thirdly, humans do not appear to dominantly encode our pulsatile stimuli as intensity formulated as mean speed (a feature that was rendered unusable in shape-change sessions by picking comparison stimuli that explicitly kept this variable constant). Note that mean speed is one of six different intensity formulations that encompass and delineate a relevant range of intensity formulations based on the observation that small changes in these intensities are correlated with poor performance (cf. Bhattacharjee et al., 2021, their Figure 2). To test the contribution of the five remaining intensity definitions, we reinterpreted our data using each of the other alternative formulations but were unable to identify a single intensity formulation that would uniquely explain the performance observed in rate-change experiments. Previous studies have portrayed mean speed as the most likely intensity code in the rodent whisker system as well as in the human fingertip (Arabzadeh et al., 2003; Gerdjikov et al., 2010; Fassihi et al., 2014). However, the confirmative present results (Bhattacharjee et al., 2021) provide direct evidence that neither mean speed nor any of the other intensity formulations alone determine the perception of pulsatile stimuli. While the six intensity formulations may cover a relevant range of formulations based on stimulus kinematics, it is presently unknown how they relate to formulations based on dynamic variables (forces and moments resulting in skin stress, particularly when considering papillary ridges rather than bulk skin). There might exist coding variables untested yet. On the same token, the biomechanics of the rodent whisker system and human fingertip ridges may be quite different (although at the microscopic level the papillary ridges of the fingertip might show stick-slip characteristics like the rodent whisker system; see Schwarz, 2016). In summary, our results using shape and rate changes confirm previous ones, and point to the possibility that local shape in pulse trains is likely encoded at greater precision than rate.

Our present aims went beyond encoding of tactile cues, i.e., investigating the storage of shape and rate-change information in memory: we were unable to find significant differences in performance between no-gap vs. gap conditions, neither using shape- nor rate-changes. This finding argues in favor of the possibility that memory storage of the encoded tactile variables happens in proportional measure to the relative role they play in encoding. Previous studies already suggested superb conservation of tactile information in memory content in humans, monkeys, and rodents: pulsatile or noise stimuli led to unimpaired performance with stimulus gaps of several seconds (Burton and Sinclair, 2000; Romo and Salinas, 2003; see for review Pleger and Villringer, 2013; Fassihi et al., 2014). However, these previous studies focused on memory storage in the framework of only one encoding scheme: typically, the contributions of either global “frequency” or global “intensity”. In view of the fact that previous studies were blind to the possible role of local kinematic variables, we hold that their results are potentially compatible with our notion of contribution of local variables for encoding and storing information. The reasons are the following: first, the studies that used sinusoidal stimulations face the problem that amplitude and frequency manipulations in sine waves concomitantly change kinematic profiles, and thus, contributions from local coding cannot be disentangled (LaMotte and Mountcastle, 1975; Arabzadeh et al., 2003). Second, a related problem exists when stimuli are drawn from Gaussian white noised trajectories (Fassihi et al., 2014): Increasing amplitude (i.e., the variance of the white noise time series), concomitantly changes the distribution of kinematic parameters contained in the stimulus. Finally, experiments using pulsatile stimulation by Romo and co-authors (e.g., Luna et al., 2005) are difficult to interpret in terms of potential usage of local codes because they used “pitch adaptation”, i.e., making pulse-shape dependent on pulse-rate impeding conclusions about the encoding of local kinematics. In conclusion, while the findings of the aforementioned studies are compatible with our study, by utilizing pulsatile stimuli we were able to disentangle local from global variables and test whether local variables are encoded and stored in memory. While the neural code to implement local coding is beyond the scope of this study, we hold it possible, given the almost instantaneous response of primary afferents to short vibrotactile events (Westling and Johansson, 1987; Laturnus et al., 2021), that spike timing might be used as an element in the tactile code (Kim et al., 2010; Mackevicius et al., 2012; Harvey et al., 2013; Weber et al., 2013; Birznieks and Vickery, 2017; Birznieks et al., 2019; Long et al., 2022). What is apparent, however, is that our results confirm the notion that local coding information is more reliable to drive vibrotactile perceptual decisions, and beyond that, point to the possibility that such encoded cues can be readily stored in short-term memory.

## Data Availability Statement

The raw data supporting the conclusions of this article will be made available by the authors, without undue reservation.

## Ethics Statement

The studies involving human participants were reviewed and approved by Ethikkommission der Universitätsklinik Tübingen. The patients/participants provided their written informed consent to participate in this study.

## Author Contributions

AB and CS: designed research, analyzed data, and wrote the article. AB: performed research. All authors contributed to the article and approved the submitted version.

## Funding

This research was supported by a grant from the Deutsche Forschungsgemeinschaft (DFG) SCHW577/14-3. Open Access funding enabled and organized by Projekt DEAL. We acknowledge support by the Open Access Publishing Fund of the University Tübingen.

## Conflict of Interest

The authors declare that the research was conducted in the absence of any commercial or financial relationships that could be construed as a potential conflict of interest.

## Publisher’s Note

All claims expressed in this article are solely those of the authors and do not necessarily represent those of their affiliated organizations, or those of the publisher, the editors and the reviewers. Any product that may be evaluated in this article, or claim that may be made by its manufacturer, is not guaranteed or endorsed by the publisher.
